# Dose-Escalated Radiation Therapy for Muscle-Invasive Bladder Cancer: A Retrospective Analysis

**DOI:** 10.1016/j.adro.2026.102156

**Published:** 2026-07-29

**Authors:** Li Chan, Anzela Anzela, Viet Do, Karen Wong, Felicia Roncolato, Joseph Descallar, Matthew Stanowski, Paul Sved, Mark Sidhom

**Affiliations:** a Liverpool and Macarthur Cancer Therapy Centres, Sydney, Australia; b Ingham Institute, Sydney, Australia; c South West Sydney clinical campuses, School of Clinical Medicine, University of New South Wales Sydney, Liverpool, New South Wales, Australia; d Department of Urology, Liverpool Hospital, Sydney, Australia; e Department of Urology, Royal Prince Alfred Hospital, Sydney, Australia; f South West Sydney Clinical School, Faculty of Medicine, University of New South Wales, Sydney, Australia

## Abstract

**Purpose:**

Patients managed with trimodal therapy (TMT) for muscle-invasive bladder cancer remain at risk of intravesical recurrences. This retrospective analysis evaluates the efficacy and safety of dose escalation compared with standard-dose radiation therapy, with differentiation between invasive and noninvasive local control.

**Methods and Materials:**

A multicenter retrospective analysis was conducted on patients with muscle-invasive bladder cancer who underwent TMT with contemporary radiation therapy techniques from 2015 to 2025. Patients in the standard-dose cohort received either 55 Gy in 20 fractions or 64 Gy in 32 fractions. In the dose-escalation cohort, a simultaneous integrated boost approach was undertaken to deliver up to 60 Gy in 20 fractions or 70 Gy in 32 fractions to the primary lesion. The effect of dose escalation compared with standard dose on 2-year local control for invasive and noninvasive disease, metastasis-free survival, overall survival, bladder preservation, and toxicity was investigated.

**Results:**

The cohort consisted of 107 patients with a median follow-up of 23 months. Dose escalation was significantly associated with improved local control of invasive disease (subdistribution hazard ratio 0.20 [0.05, 0.89], *P* = .035) compared with standard dosing, after adjusting for T stage. There were no differences in local control for noninvasive disease (*P* = .8), metastasis-free survival (*P* = .8), and overall survival (*P* = .5). Both groups tolerated radiation therapy well, with no difference in grade 2 or greater genitourinary and gastrointestinal toxicity (*P* = .8, *P* = .9, respectively). No patients receiving dose escalation required salvage cystectomy for invasive disease recurrence, compared with 2 patients who received standard dosing.

**Conclusions:**

Preliminary evidence suggests efficacy of dose escalation with good tolerability, high rates of bladder preservation, and potentially lower rates of invasive recurrences compared with standard-dose radiation therapy. This study’s findings support the need for further prospective studies to evaluate dose escalation as a technique to maximize the efficacy of TMT.

## Introduction

Bladder cancer is the 11th most prevalent malignancy in Australia, with 5-year mortality rates demonstrating an upward trend.^1^ Muscle-invasive bladder cancer (MIBC) constitutes 20% to 30% of bladder cancer cases and is associated with significant morbidity and mortality.^2^ Trimodal therapy represents an alternative treatment to radical cystectomy for select patients with MIBC and is supported by national and international guidelines.^3^, ^4^, ^5^, ^6^ This approach consists of maximal transurethral resection of bladder tumor (TURBT) followed by concurrent chemotherapy and radiation therapy. Radiation therapy regimens commonly used are 55 Gy in 20 fractions or 64 Gy in 32 fractions as a uniform dose to the entire bladder.^5^^,^^7^ In cases where chemotherapy is contraindicated, radiation therapy can be delivered alone, but with reduced efficacy.^8^

Patients who undergo trimodal therapy remain at risk of developing intravesical recurrences.^9^ Notably, invasive recurrences confer a significantly poorer prognosis than noninvasive recurrences, and salvage cystectomy remains the standard of care for patients with invasive relapses who are fit enough for surgery.^9^^,^^10^ A recent mapping analysis demonstrated that the majority of invasive recurrences arise in the proximity of the original muscle-invasive tumor site,^11^ indicating that standard-dose radiation therapy may be inadequate to eradicate residual malignant foci following transurethral resections.^12^^,^^13^

There has been growing interest in the escalation of radiation therapy dose to the primary bladder tumor site to improve oncologic outcomes. This entails the delivery of higher doses to the gross tumor of up to 60 Gy in the 20 fractions or 70 Gy in the 32 fractions schedule.^5^ Recent studies have demonstrated that dose escalation is feasible and safe, with low incidences of grade 3 or higher acute and late genitourinary (GU) and gastrointestinal (GI) toxicities.^14^^,^^15^

Existing literature comparing outcomes between dose escalation and standard radiation therapy for patients with MIBC remains limited, and the results are conflicting.^15^, ^16^, ^17^ In addition, none of the studies to date have reported on the differences in outcomes between localized invasive and noninvasive recurrences. This study aimed to conduct a retrospective analysis to investigate the efficacy and safety of dose-escalation radiation therapy to the bladder compared with standard dosing, while distinguishing between invasive and noninvasive recurrence outcomes.

## Methods and Materials

A retrospective analysis was conducted on data from 3 oncology centers between March 2015 and May 2025. This time period was selected as it encompassed modern treatment techniques for patients with bladder cancer by incorporating image guidance with daily cone beam computed tomography (CBCT) before radiation therapy. The inclusion criteria were patients with MIBC who underwent TURBT followed by radiation therapy, with or without concurrent chemotherapy. The cohort included patients with node-positive disease, provided they exhibited no evidence of distant metastasis and were managed with curative intent. Patients with metastatic disease or those who received treatment with palliative intent were excluded from this cohort. Other variables recorded include patient age, gender, performance status, smoking history, tumor variant, number of tumor foci, presence of hydronephrosis, fractionation schedule (hypofractionated or conventional), and details of chemotherapy administration, including the type of systemic agent. Ethics approval for this study was provided by the South Western Sydney Local Health District Ethics Committee (2025/ETH01071).

**Figure 1:**
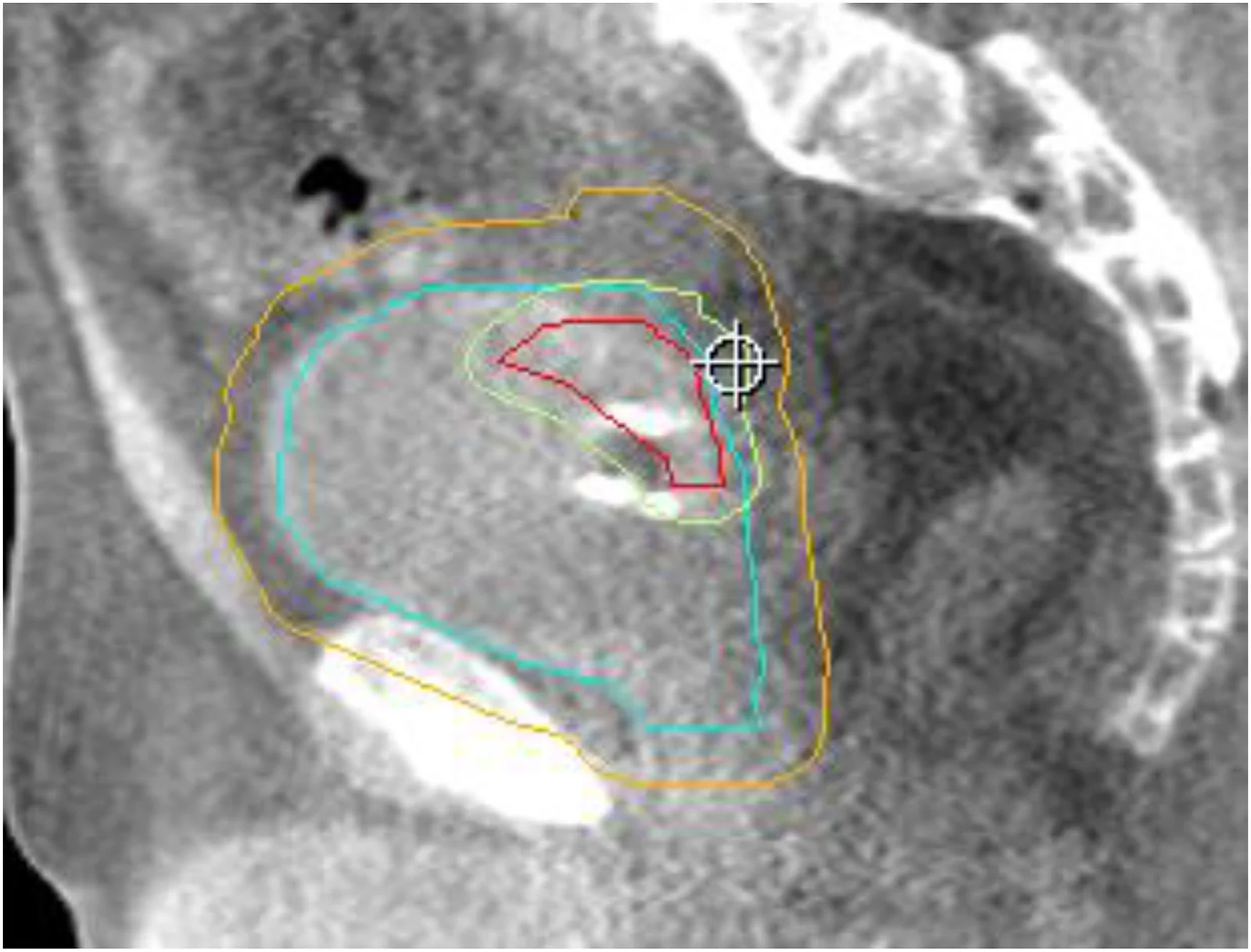
CBCT demonstrating the bladder is within the PTV. CTV whole bladder (teal), PTV whole bladder (orange), GTV boost (red), PTV boost (green). *Abbreviations:* CBCT = cone beam CT; CT = computed tomography; CTV = clinical target volume; GTV = gross target volume; PTV = planning target volume.

All patients underwent computed tomography (CT) simulation for treatment planning with an empty bladder to optimize target delineation and minimize organ motion. In select patients, planning magnetic resonance imaging (MRI) and staging fluorodeoxyglucose positron emission tomography (FDG-PET) were fused to enhance target definition. The planning CT and MRI served as the primary imaging for bladder target volume delineation, while the FDG-PET provided supplementary metabolic information for target definition. Patients were instructed to void before PET acquisition to minimize intravesical tracer activity. All treatments were delivered via volumetric-modulated arc therapy. Daily CBCT was routinely utilized from 2016 onward for image-guided treatment verification (Fig. 1). If excessive bladder volume was detected before radiation therapy, patients were instructed to void, followed by a repeat CBCT to ensure that the bladder size was appropriate.

**Figure 2:**
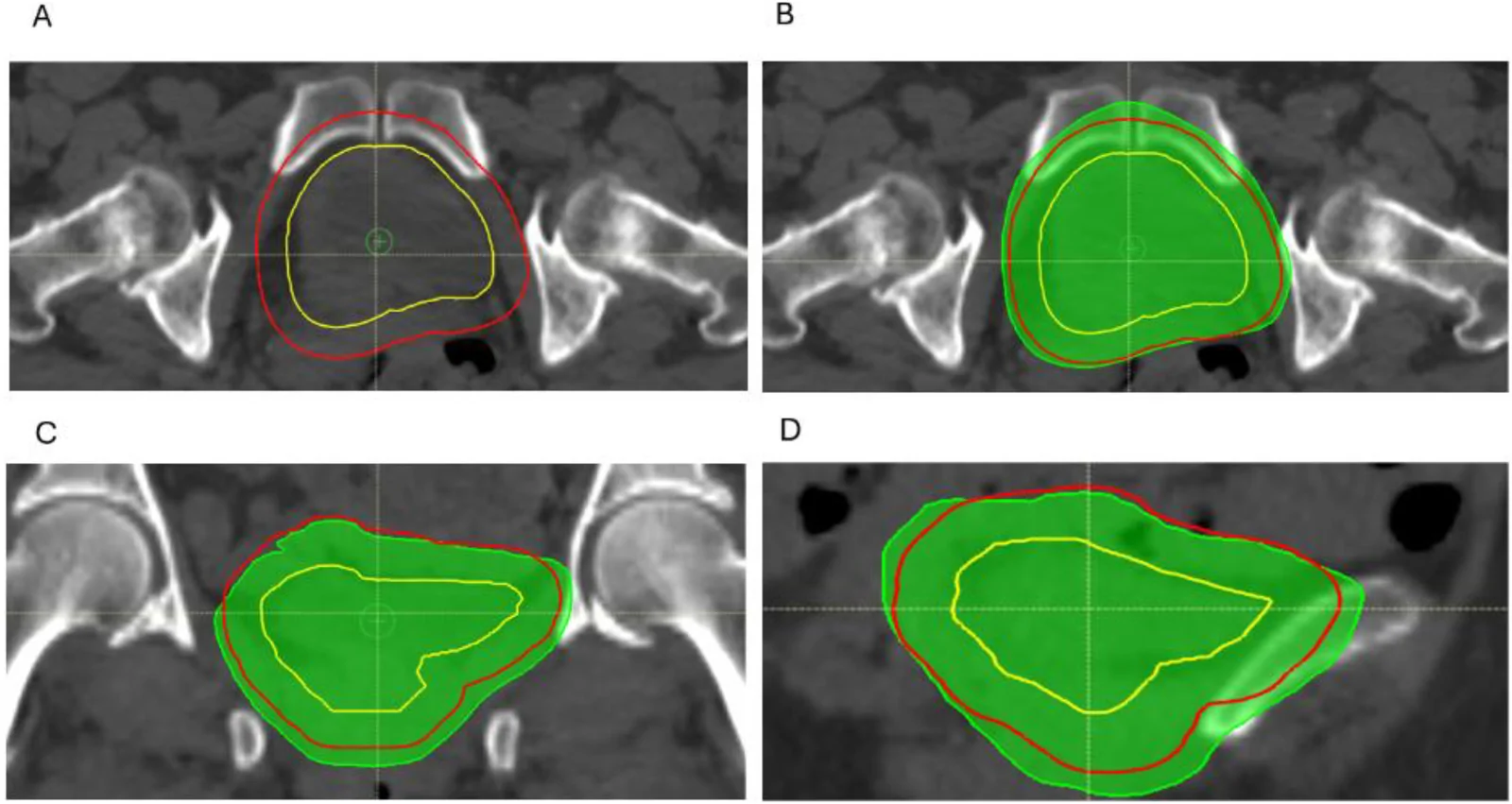
CT simulation (A) and dosimetry images (axial [B], coronal [C] and sagittal [D]) of a standard-dose radiation therapy plan. The CTV is delineated in yellow, and the PTV is in red. The green represents the 95% isodose wash. *Abbreviations:* CT = computed tomography; CTV = clinical target volume; PTV = planning target volume.

**Figure 3:**
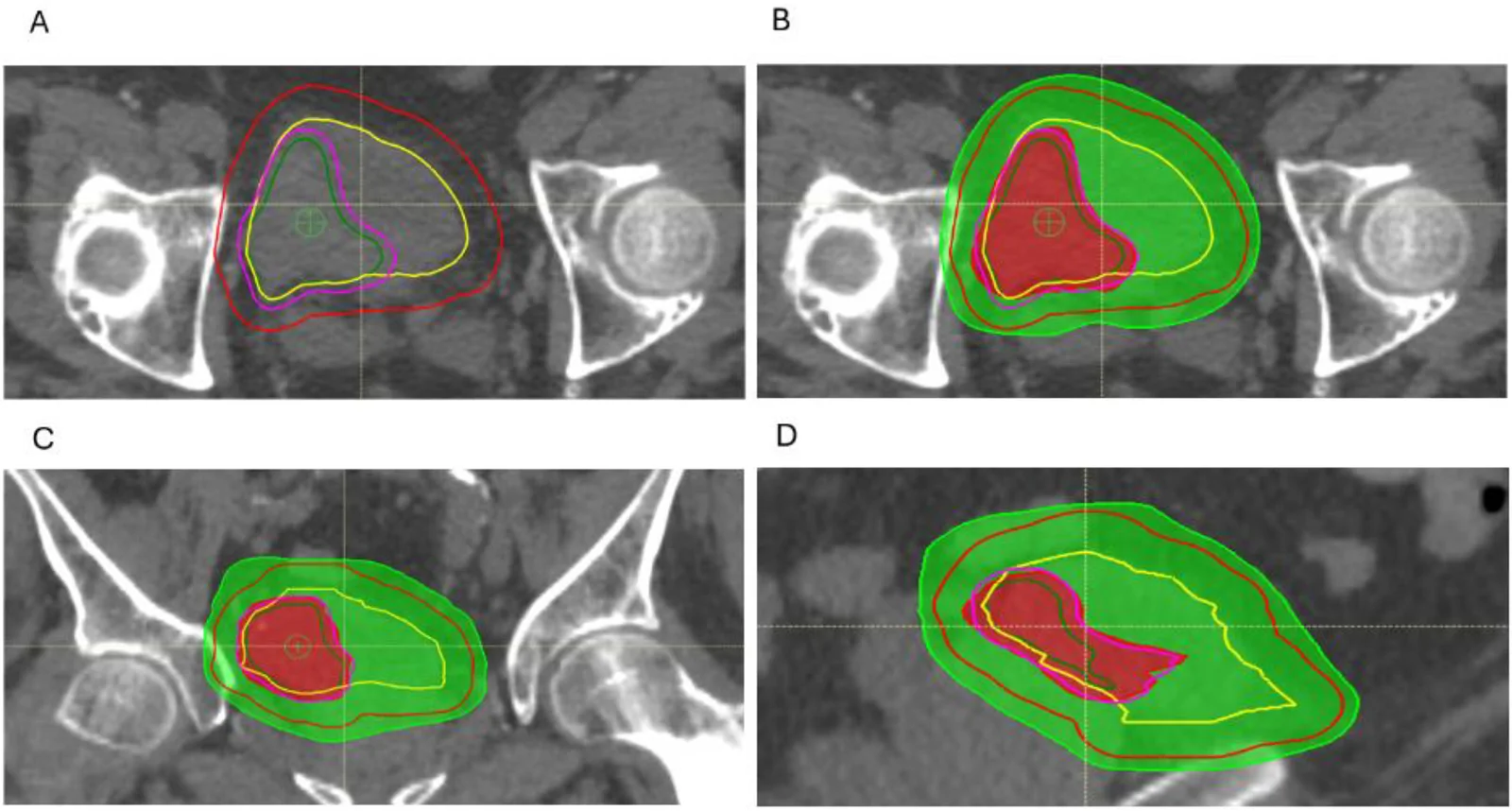
CT simulation image of a dose-escalation radiation therapy plan (A), the GTV boost is delineated in dark green and PTV boost is in pink. The remaining 3 images represent the axial (B), coronal (C) and sagittal (D) views of the dosimetry plan. The red dose wash represents the high-dose boost region. *Abbreviations:* CT = computed tomography; GTV = gross target volume; PTV = planning target volume.

In the standard-dose cohort, the prescribed radiation regimens consisted of either 55 Gy in 20 fractions or 64 Gy in 32 fractions to the entire bladder. The entire bladder was delineated as the clinical target volume, followed by a 1 cm expansion to the planning target volume (Fig. 2). For the dose-escalation group, a simultaneous integrated boost approach was undertaken to deliver up to 60 Gy in 20 fractions or 70 Gy in 32 fractions. The boost volume was delineated by contouring the gross tumor on CT, aided by the TURBT operation report, staging MRI, and FDG PET, if required. This was followed by a 5 mm isotropic expansion to create the planning target volume (Fig. 3).

Pelvic nodal radiation was delivered for all patients with nodal metastasis. For the 20-fraction cohort, prescribed doses were 50 Gy to the involved nodes and 45 Gy to the elective pelvic nodes. In the 32-fraction cohort, the corresponding doses were 60 Gy and 52 Gy, respectively. The elective nodal clinical target volume encompassed the bilateral common iliac, external iliac, internal iliac, and obturator nodal regions. Elective nodal irradiation was not performed for node-negative patients. Concurrent systemic therapy consisted of either cisplatin or a combination of mitomycin and 5-fluorouracil.

Following completion of treatment, all patients underwent surveillance cystoscopies at 3 to 6 monthly intervals, as determined by the treating urologist. CT of the chest, abdomen, and pelvis was conducted every 6 months within the first 2 years after completion of treatment, followed by yearly scans thereafter. Patients with localized invasive recurrences were referred for salvage cystectomies if deemed fit for surgery. GU and GI toxicity were recorded according to the National Cancer Institute Common Terminology Criteria for Adverse Events.^18^ Treatment-related adverse events up to 3 months after the end of treatment were categorized as acute toxicity.

### Statistical analysis

The study endpoints included 2-year local control, metastasis-free survival, and overall survival. For the assessment of local control, recurrences were classified as invasive and noninvasive recurrences. This was defined as the occurrence of the event via cystoscopic evaluation. For patients who did not undergo cystoscopy due to either medical comorbidities or patient preference, recurrent bladder lesions detected on CT imaging were considered invasive recurrences following multidisciplinary team review. Metastasis-free survival was defined as the interval free from the occurrence of nodal or metastatic disease. All time-to-event analyses were calculated from the first day of radiation to the date of the respective event.

For local control and metastasis-free survival, cumulative incidence functions were estimated with death from any cause as a competing event. Univariable and multivariable analyses were conducted using Fine-Gray subdistribution hazard models. For overall survival, the Kaplan-Meier method was used to estimate survival probabilities, and between-groups differences were assessed using the log-rank test. The Cox proportional hazards regression model was used for univariable and multivariable analysis. The following covariates were assessed: dose escalation, T stage, unifocal versus multifocal disease, presence of hydronephrosis, use of concurrent chemotherapy, variant histology, and fractionation regimen

Multivariable models were constructed to estimate the adjusted effect of dose escalation after controlling for potential confounders. Covariates were selected based on clinical relevance rather than statistical significance on univariate analysis. For local control and metastasis-free survival, the limited number of events required restriction of the multivariable Fine-Gray model to 2 prespecified covariates (dose escalation and T stage) to avoid model overfitting. T stage was selected as the primary confounder given its established prognostic significance for local recurrence and metastasis-free survival outcomes in MIBC.^19^ For overall survival, the multivariable Cox regression model included all covariates. Hazard ratios (HR) and subdistribution hazard ratios (SHR) were reported with 95% CIs, and *P* values <.05 were considered statistically significant. All calculations were performed using Stata (StataCorp).

## Results

Between March 2015 and May 2025, 107 consecutive patients treated for MIBC with curative intent were identified. Of these, 39 patients received dose-escalated radiation therapy, while the remaining 68 received standard dosing. The median follow-up duration was 23 months (range, 3-104 months). Approximately half of the cohort (51%) did not receive chemotherapy, predominantly due to poor performance status and significant comorbidities. All but 1 patient underwent daily CBCT for treatment verification, as they underwent treatment before 2016. The relevant patient, treatment, and tumor characteristics of the cohort are displayed in Table 1.

**Table 1:** Baseline cohort characteristics^a^

	Standard	Dose escalation	Total
n	68	39	107
Gender			
M	49 (72%)	32 (82%)	81 (76%)
F	19 (28%)	7 (18%)	26 (24%)
ECOG			
0-1	31 (46%)	22 (56%)	53 (50%)
2	30 (44%)	14 (36%)	44 (41%)
3	7 (10%)	3 (8%)	10 (9%)
T stage			
2	57 (84%)	36 (92%)	93 (87%)
3	11 (16%)	3 (8%)	14 (13%)
Nodal stage			
Negative	58 (85%)	36 (92%)	94 (88%)
Positive	10 (15%)	3 (8%)	13 (12%)
Tumor variant			
N	54 (79%)	27 (69%)	81 (76%)
Y	13 (19%)	9 (23%)	22 (21%)
N/A	1 (2%)	3 (8%)	4 (3%)
Hydronephrosis			
Y	19 (28%)	4 (10%)	23 (22%)
N	49 (72%)	35 (90%)	84 (78%)
Tumor foci			
Single	44 (65%)	39 (100%)	83 (78%)
Multiple	24 (35%)	0 (0%)	24 (22%)
Concurrent chemo			
Y	30 (44%)	22 (56%)	52 (49%)
N	38 (56%)	17 (44%)	55 (51%)
Fractionation			
Conventional	9 (13%)	1 (3%)	10 (9%)
Hypofractionation	59 (87%)	38 (97%)	97 (91%)

a *Abbreviations:* ECOG = Eastern Cooperative Oncology Group Status; n = sample size; M = male; F = Female; T = Tumour stage; Y = yes; N = no; N/A = not available.

### Local control

**Figure 4:**
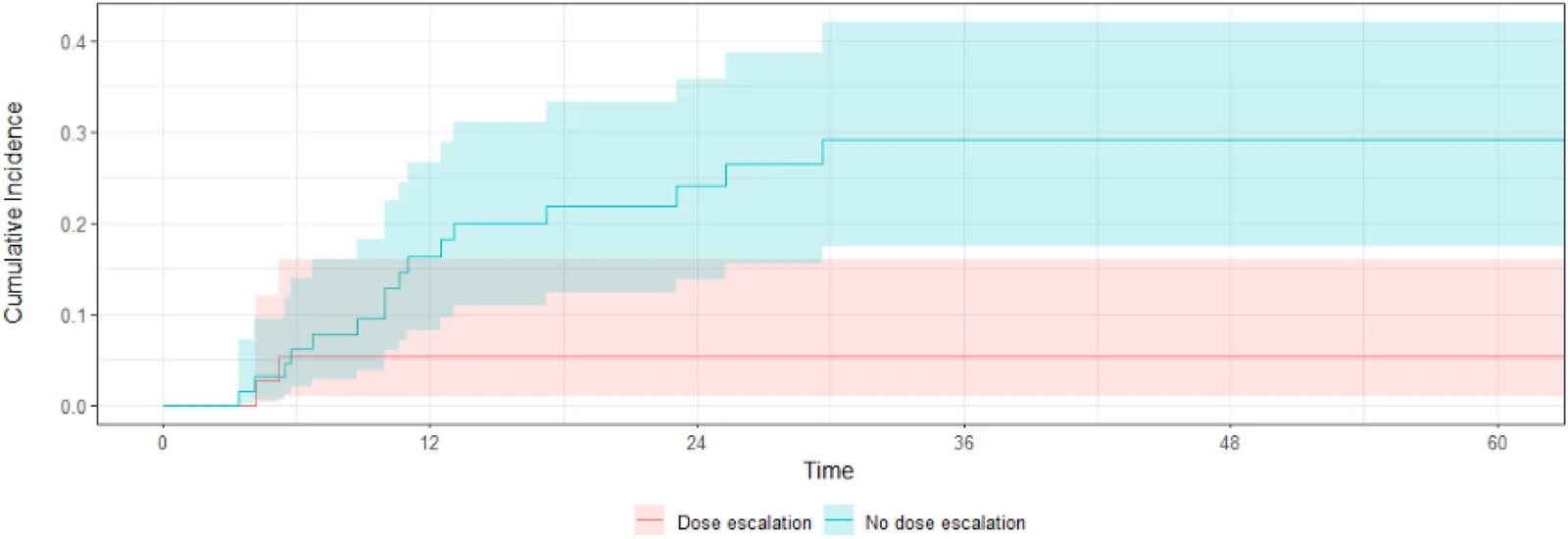
Cumulative incidence of invasive local recurrence by treatment group.

Within the dose-escalation cohort, 2 patients (5.1%) developed invasive recurrence, whereas 3 (7.7%) had noninvasive recurrence. In contrast, there were 16 invasive (23.5%) and 7 noninvasive recurrences (10.3%) for the standard-dose group. The 2-year cumulative incidence of invasive recurrence was 5.5% in the dose-escalation group and 27.5% in the standard-dose group (Fig. 4). On univariable Fine-Gray analysis, there was a statistically significant lower risk of invasive recurrence for patients who received dose escalation compared with standard dosing (SHR 0.21 [0.05, 0.94], *P* = .04). On multivariable analysis adjusting for T stage, dose escalation remained significantly associated with a lower risk of recurrence (SHR 0.20 [0.05, 0.89], *P* = .035). No other covariates, including concurrent chemotherapy, histology subtype, number of tumor foci, fractionation regimen, tumor stage, and presence of hydronephrosis, reached statistical significance on univariable analysis.

For noninvasive recurrence, the 2-year cumulative incidence was 6.7% and 9.9% for the escalated-dose and standard-dose group, respectively (Fig. E1). On univariable analysis, dose escalation was not significantly associated with noninvasive recurrence (SHR 0.88 [0.23-3.33], *P* = .98). This remained nonsignificant (*P* = .79) after multivariable analysis. No other covariates reached significance on univariable analysis.

In regard to the site of recurrence for the dose-escalation cohort, 1 of 2 invasive recurrences (50%) occurred within the boost field, while the other occurred outside the boosted region. None of the 3 noninvasive recurrences occurred within the boost field. The pattern of recurrence for the cohort is summarized in Table 2.

**Table 2:** Pattern of recurrence by site and cohort

Site of recurrence	Invasive (N)	Noninvasive (N)
Dose-escalation cohort		
Within boost field	1	0
Outside boost field	1	3
Standard-dose cohort		
Within original tumor site	14	2
Outside original tumor site	2	5

There were 3 recurrences within the standard-dose cohort that were detected via imaging rather than cystoscopy, as these patients had medical comorbidities precluding cystoscopy. These patients underwent contrast-enhanced CT of the abdomen and pelvis, which demonstrated enlargement of the primary bladder mass lesion. These were classified as presumed invasive recurrence based on clinical context and multidisciplinary team consensus. All recurrences in the dose-escalation cohort were confirmed via cystoscopy.

### Metastasis-free survival

In the dose-escalation group, 8 patients (20.5%) developed nodal or distant metastasis, while there were 22 (32.4%) in the standard dose. The 2-year cumulative incidence of metastasis was lower within the dose-escalation group at 21.1% as compared with 32.3% in the standard group (Fig. E2). However, this difference did not translate into significance on univariable analysis (*P* = .79) as well as multivariable analysis (*P* = .75). Other covariates were also not significant on univariable analysis.

### Overall survival

The 2-year overall survival rates for the cohort who received dose escalation and standard dose were 71.1% and 64.4%, respectively. On univariable analysis, there was no significant difference in overall survival outcomes between the 2 groups (*P* = .5) (Fig. E3). Absence of chemotherapy was associated with worse overall survival (HR 2.45 [1.25-4.81], *P* < .01), whereas patients without hydronephrosis had improved overall survival (HR 0.4 [0.2, 0.8], *P* < .01). These variables remained significant on multivariable analysis (HR 2.83 [1.42, 5.63], *P* < .01, HR 0.34 [0.16, 0.71], *P* < .01).

### Bladder preservation

There were high rates of bladder preservation within the cohort (Fig. E4). In the standard treatment group, 2 patients (2.9%) required salvage cystectomy for invasive disease recurrence. Only 1 patient (2.5%) from the dose-escalation group underwent salvage cystectomy for recurrent noninvasive disease that was refractory to Bacillus Calmette-Guerin. The majority of patients with invasive relapses did not proceed to a salvage cystectomy as they were not fit for surgery at the time of recurrence.

### Toxicity

There were 15 patients (22.1%) who developed grade 2 or greater (2+) GU toxicity in the standard dose, compared with 7 (17.9%) in the dose-escalation group. For GI toxicity, 5 patients (7.4%) in the standard group and 2 (5.1%) in the dose-escalation group developed G2+ toxicity. Grade 3 toxicity was only observed in 2 patients (2.9%), both of whom were in the standard group. On analysis, there was no significant difference for G2+ GU or GI toxicity between groups (*P* = .8 and *P* = .9, respectively). No patients required early cessation of treatment due to toxicity.

## Discussion

Our study reports higher rates of local control of invasive disease for patients who underwent dose-escalated radiation therapy for MIBC, compared with patients who received standard doses of radiation therapy. No significant differences were observed for local control in noninvasive disease, metastasis-free survival, or overall survival. Importantly, dose escalation was not associated with increased G2+ toxicity, indicating a favorable safety profile.

There has been an increasing interest in dose escalation for MIBC to improve outcomes, although existing data are conflicting. The RAIDER phase 2 trial reported numerically higher 2-year locoregional control rate with dose escalation (74% vs 66%), although invasive locoregional control rates were similar (83% vs 80%).^15^ Notably, the RAIDER trial reported invasive locoregional control as a composite endpoint encompassing both invasive bladder recurrences and regional nodal failure, rather than examining localized intravesical invasive recurrence as a distinct endpoint.^15^ Murthy et al,^14^ who differentiated between superficial and muscle-invasive local recurrences, reported similar local recurrence rates for both outcomes between dose escalation and standard dosing. In contrast, Chang et al^16^ reported improved locoregional control with dose escalation, though the distinction between noninvasive recurrences and invasive recurrences was also not investigated.

In the present study, dose escalation was associated with a significant improvement in local control of invasive disease. By examining localized invasive recurrence as a separate endpoint that is distinct from regional nodal failures, the present study may have captured a clinically meaningful outcome that is diluted in composite locoregional endpoints used in prior studies. This distinction is clinically relevant, as patients with noninvasive recurrences may be managed with bladder-preserving approaches such as TURBT and intravesical therapies, whereas those with invasive recurrences typically require salvage cystectomy, which is associated with higher complication rates as well as poorer quality of life outcomes.^9^^,^^10^^,^^20^ Therefore, by reducing invasive recurrences, dose escalation may confer a significant benefit not only for oncologic outcomes but also for quality of life. Nonetheless, these findings should be interpreted with caution, given the limited sample size. Larger prospective studies incorporating this specific endpoint are needed to confirm whether dose escalation translates into an independent oncologic and quality of life advantage.

The absence of metastasis-free survival and overall survival benefit with dose escalation in our study is in accordance with existing evidence. Neither the RAIDER trial nor a population-based analysis demonstrated a progression-free survival or overall survival benefit with dose escalation.^15^^,^^17^ This is likely attributable to high rates of occult micrometastasis at diagnosis, which are undetectable via routine staging imaging such as FDG PET/CT.^21^^,^^22^ Consequently, dose escalation may be insufficient to reduce the risk of distant metastasis or improve overall survival. Interpretation in the present study is further limited by the higher proportion of adverse prognostic features in the standard-dose cohort, including T3 disease, nodal involvement, and hydronephrosis, all of which independently influence survival outcomes.^23^^,^^24^

In the analysis of covariates, hydronephrosis was significantly associated with inferior overall survival. This has similarly been reported in prior studies, demonstrating its correlation with increased risk of extravesical extension and nodal metastasis.^24^ The observed association between concurrent chemotherapy and improved overall survival is not fully concordant with existing evidence. Concurrent chemoradiation therapy as part of trimodality treatment for MIBC is the standard of care based on the BC2001 trial, which demonstrated improved locoregional control and metastasis-free survival with systemic therapy, but only a modest and nonsignificant overall survival benefit.^25^ While chemotherapy’s radiosensitizing effect could reduce the incremental benefit of dose escalation, the low and variable chemotherapy rates across both cohorts in the present study limit the ability to isolate the independent effect of dose escalation. Further investigation is required to determine whether dose escalation provides an additive benefit alongside optimal chemoradiation therapy or primarily benefits chemotherapy-ineligible patients.

The present study did not demonstrate a significant difference in local control between standard and hypofractionated regimens, contrasting with a meta-analysis, which reported superior invasive locoregional control with hypofractionation.^26^ The present study also did not report a significant difference between unifocal and multifocal disease, despite multifocal disease being a recognized independent predictor of worse local control.^19^ These discrepancies likely reflect the limited power to detect differences due to imbalanced group sizes, with the majority of the cohort undergoing hypofractionation, and patients with multifocal disease were excluded from dose escalation.

Regarding treatment-related toxicity, this study’s findings demonstrate that dose escalation with a simultaneous integrated boost approach is a safe treatment, with no difference in grade 2 or greater GU and GI toxicity with the standard fractionation regimens. The technical complexity of bladder radiation therapy is notable due to the organ’s mobility and variability in shape and size. Dose escalation, therefore, confers an increased risk of GU and GI toxicity due to the greater potential of high-dose irradiation to surrounding organs at risk.^27^ Nevertheless, our study findings of low rates of toxicity are concordant with other trials,^15^^,^^27^ supporting the safety of dose escalation when combined with daily pretreatment imaging. However, the short median follow-up period of this study precludes assessment of long-term toxicity, which may manifest beyond the current observation window.

The treatment paradigm for MIBC is rapidly evolving with emerging systemic therapies that may redefine the role of radiation. Recent data from the Keynote-905 trial demonstrated significant pathologic complete response rates with neoadjuvant enfortumab vedotin plus pembrolizumab, prompting discussions regarding potential therapy de-escalation.^28^ In addition, ongoing trials such as the SWOG S2427 and NRG GU015 are investigating whether circulating tumor DNA clearance could guide de-escalation of local treatment in responding patients.^29^^,^^30^ Although the present study suggests potential benefits of dose escalation for local control in the current treatment paradigm, future research must balance maximizing local control with emerging opportunities for treatment personalization and de-escalation.

This study is subject to several limitations. There was an imbalance in patient, treatment, and tumor factors between the 2 cohorts, including T and N stage and chemotherapy rates, that could have impacted the analysis. In addition, several other key confounding variables were not evaluated, such as the extent of TURBT and the presence of carcinoma in situ.^6^ Additionally, a small proportion of patients were classified as having invasive recurrence based on imaging rather than cystoscopy. Therefore, the results of this study are hypothesis-generating, and prospective randomized studies are required to assess the efficacy of dose escalation compared with standard fractionation in patients with MIBC before it can be considered as standard of care.

## Conclusion

This study demonstrates that dose-escalated radiation therapy for MIBC is well tolerated, and potentially results in higher rates of local control of invasive disease when compared with standard fractionation. However, no benefit was observed for noninvasive local control, metastasis-free survival, and overall survival. Dose escalation is feasible and associated with an acceptable safety profile, supporting further investigation of its use as a therapeutic strategy for patients with MIBC.

## Disclosures

The authors declare that they have no known competing financial interests or personal relationships that could have appeared to influence the work reported in this paper.

## Appendix Supplementary materials

Supplementary Figures Image, application 1

## Footnotes

Supplementary material associated with this article can be found in the online version at doi:10.1016/j.adro.2026.102156.
