# Supplementary material for: Dose-Escalated Radiation Therapy for Muscle-Invasive Bladder Cancer: A Retrospective Analysis
Source: Adv Radiat Oncol. 2026 Jul 29;11(12):102156. doi: 10.1016/j.adro.2026.102156 (PMC13571918; doi:10.1016/j.adro.2026.102156)
Supplement: Supplementary file 1 [file mmc1.docx]

**Supplementary Figures**

Supplementary Figure 1: Cumulative incidence of non-invasive local recurrence by treatment group

Supplementary Figure 2: Cumulative incidence of metastasis by treatment group

Supplementary Figure 3: Overall survival of the cohort

Supplementary Figure 4: Bladder preservation rates of the cohort
